# MAP GERIATRICS CLERKSHIP LEARNING OBJECTIVES WITH GERIATRICS 5MS

**DOI:** 10.1093/geroni/igad104.2423

**Published:** 2023-12-21

**Authors:** Huai Cheng

**Affiliations:** University of Pittsburgh, Pittsburgh, Pennsylvania, United States

## Abstract

**Background:**

Minimum Geriatrics Competencies (MGC) are used to guide teaching geriatrics for medical students in a required geriatrics clerkship since 2011. These learning objectives can be mapped to Entrustable Professional Activities (EPA). Since 2017, Geriatrics 5Ms (mind, mobility, medication, multi-complexities, and what matters most) was developed to guide teaching geriatrics for medical students and to promote a Geriatrics 4Ms Age-friendly health care system as well. The aim of this study is to examine whether an MGC-based geriatrics clerkship is consistent with geriatrics 5Ms by mapping its learning objectives with geriatrics 5Ms.

**Methods:**

This geriatrics clerkship is a two-weeks required rotation for all fourth-year medical students. It includes didactic classroom teaching and clinical rotation in different geriatrics practice, palliative care, and physical medicine rehabilitation. This clerkship has a total of 50 learning objectives with corresponding MGC teaching methods/strategies, assessment, and evaluation. The author will map each learning objective with Geriatrics 5Ms. Results 13 learning objectives are mapped to “Mind”. 9 learning objectives are mapped to “Mobility”. 10 learning objectives are mapped to “Medications”. 14 learning objectives are mapped to “multi-complexity”. 6 learning objectives are mapped to “Matters most”. 12 learning objectives are not mapped to any of geriatric 5Ms. Conclusion and discussion The majority of 50 learning objectives in a required geriatrics clerkship can be mapped to geriatrics 5Ms. This MGC-based learning objective can be adopted to teach medical students geriatric 5Ms and geriatric 4M Age-friendly health care system. Take-home message MGC-based learning objectives are aligned with geriatrics 5Ms.

